# *Mycoplasma hyopneumoniae* modulates ciliary function and epithelial integrity in air-liquid interface porcine respiratory epithelial cells (ALI-PRECs)

**DOI:** 10.1128/spectrum.01998-25

**Published:** 2026-06-18

**Authors:** Ana F. Castillo-Espinoza, Rahul K. Nelli, Juan C. Mora-Díaz, Danyang Zhang, Rolf Rauh, Bala M. Reddi, Apoorva Saxena, Ning-Chieh Twu, Raquel Espin-Palazón, Luis G. Giménez-Lirola

**Affiliations:** 1 Department of Veterinary Diagnostic and Production Animal Medicine, College of Veterinary Medicine, Iowa State University 1177, Ames, Iowa, USA; 2 Department of Statistics, College of Liberal Arts and Sciences, Iowa State University 1177, Ames, Iowa, USA; 3 Tetracore Inc. 248666 , Rockville, Maryland, USA; 4 Department of Genetics, Development and Cell Biology, Iowa State University 1177, Ames, Iowa, USA; University of Manitoba, Winnipeg, Canada

**Keywords:** *Mycoplasma hyopneumoniae*, air-liquid interface culture, porcine respiratory epithelial cells, ciliary motility, epithelial disruption, pathogenesis, gene expression regulation, host-pathogen interactions

## Abstract

*Mycoplasma hyopneumoniae* (*Mhp*), the causative agent of enzootic pneumonia, disrupts mucociliary clearance by adhering to porcine ciliated airway cells, contributing to chronic respiratory disease. Traditional submerged cell cultures lack airway-like polarity and structural complexity, limiting *Mhp in vitro* studies. This study utilized a porcine air-liquid interface porcine respiratory epithelial cell (ALI-PREC) model to investigate *Mhp* infection dynamics, cytopathic effects (CPEs), and host-specific responses. Primary tracheal epithelial cells isolated from three 6-week-old pigs were cultured under air-liquid interface conditions for 4 weeks to form differentiated pseudostratified, ciliated epithelium. ALI-PRECs were inoculated with *Mhp* strain 232 at 10^5.84^, 10^6.84^, and 10^7.84^ CCU/mL for 2 or 5 h and monitored for 144 h. Quantitative microscopy assessed total particle count, area, average size, and percentage area covered by particles, normalized against mock controls. *Mhp* adhered within 2 h, inducing CPE (cell rounding, clustering, and detachment) and reducing ciliary activity in a dose- and time-dependent manner. *Mhp* DNA was detected in the epithelium by 24 h post-inoculation (hpi) and in subnatants at higher doses, demonstrating epithelial barrier disruption. Ciliary beating persisted in some replicates, suggesting interindividual variability in host responses. Friis medium had no detectable impact on ALI-PRECs, validating its suitability for infection studies. Gene expression analysis revealed downregulation of ciliary motility genes (*ROPN1L*, *LRRC51*, *CEP162*, and *LRRC6*) at 72 hpi and upregulation of intercellular junction genes (*CLDN1*, *CDH1*, and *CTNND1*) by 120 hpi, suggesting a wound healing response. The ALI-PREC model effectively mirrors key aspects of *Mhp* pathogenesis, providing a robust platform for studying host-pathogen interactions and identifying therapeutic targets.

**IMPORTANCE:**

Despite its economic impact on pigs, the mechanisms by which *Mycoplasma hyopneumoniae* (*Mhp*) causes enzootic pneumonia remain poorly understood. Early infection events (bacterial exposure and adherence) are hard to study *in vitro* because traditional cell cultures lack airway complexity and cellular diversity. We developed an *in vitro* model replicating porcine airway structure and cell populations. Using this model, we showed that *Mhp* rapidly adheres to airway cells and disrupts ciliary function and epithelial integrity in a dose- and time-dependent manner. These effects coincide with altered expression of genes governing ciliary motility and cell-cell junctions. Importantly, our model revealed natural variability in cytopathic responses among pigs, indicating that host-specific factors influence disease progression. These insights may guide personalized strategies for preventing and treating swine respiratory infections.

## INTRODUCTION

*Mycoplasma hyopneumoniae* (*Mhp*) has been extensively studied as the causative agent of porcine enzootic pneumonia and a key contributor to the porcine respiratory disease complex (1). *Mhp* infection induces chronic bronchopneumonia, adversely impacting growth rates (2), predisposing pigs to co-infections (3), and increasing mortality in wean-to-finish operations (4). The initial *Mhp* adherence to host tissues is a critical and complex process mediated primarily through bacterial adhesins and porcine airway ligands, including glycosaminoglycans on ciliated cells and extracellular matrix (ECM) components (1, 5–7). Lacking biosynthetic capabilities, *Mhp* relies on host-derived nutrients, producing significant metabolic bioproducts that deplete host resources and degrade the ECM, contributing to airway damage (8, 9). While *Mhp* colonization of cilia is known to cause ciliostasis and cilia loss (5, 10), the specific structural and molecular changes in porcine cells upon infection remain unexplored. Recent studies in *Mhp*-infected swine epithelial monolayers indicate that the downregulation of ciliary genes may drive this ciliostasis (11), and a reversible disruption of epithelial integrity mediated by tight junctions has also been reported in bronchial cells (12), suggesting multiple mechanisms by which *Mhp* compromises airway integrity.

To evaluate *Mhp* attachment and early innate immune response at the porcine airway epithelium, it is important to establish a cell culture model that mimics an *in vivo* animal model, offering an ethically sound (3R principles: replacement, reduction, and refinement) and reductionist platform (12, 13). However, traditional two-dimensional submerged culture models fail to accurately mimic the *in vivo* respiratory microenvironment, limiting their effectiveness in studying host-*Mhp* interactions comprehensively (14). In contrast, porcine-derived primary respiratory epithelial cells (PRECs) cultured at the air-liquid interface (ALI), a three-dimensional approach, polarized architecture that closely resembles the native airway structure, making air-liquid interface porcine respiratory epithelial cell (ALI-PREC) a suitable model for respiratory pathogen studies (13, 15, 16), including those focused on *Mhp* (12).

This study investigates the kinetics of *Mhp* strain 232 infection in ALI-PRECs. The susceptibility of ALI-PRECs to *Mhp* was initially assessed, confirming its dependence on the dose and time point, as well as the influence of the exposure time to the bacteria. A detailed kinetic study on infection progression, employing three biological replicates and extended time points, revealed that *Mhp* modulates ciliary function and epithelial integrity, which is evidence of localized cytopathic damage and transcriptional regulation of genes related to ciliary motility and intercellular junctions.

## RESULTS

### Rapid adherence of *Mhp* to ALI-PRECs

**Fig 1:**
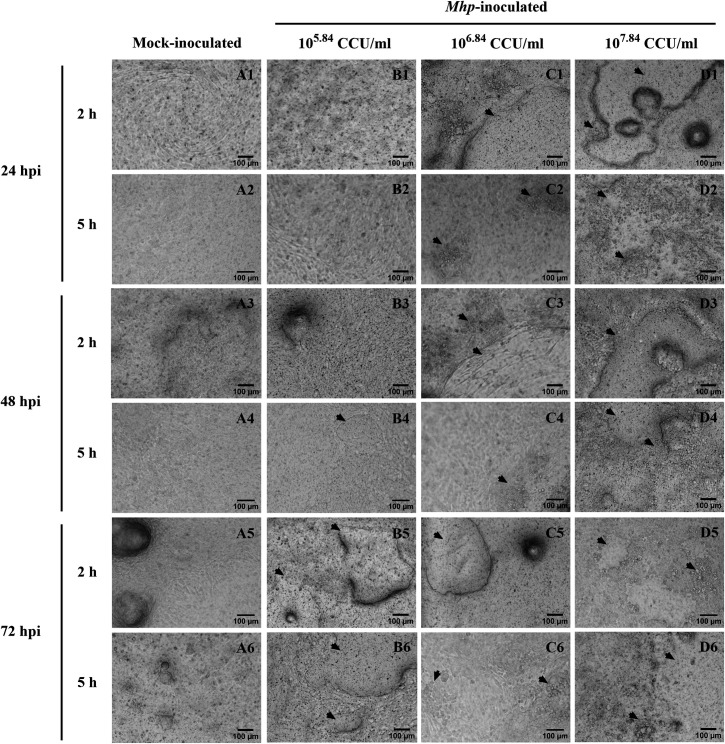
Temporal assessment of the cytopathic damage in ALI-PRECs following *Mhp* exposure. ALI-PRECs were mock-inoculated with infection medium or exposed to *Mhp* at doses of 10^5.84^, 10^6.84^, and 10^7.84^ CCU/mL for 2 or 5 h. Cytopathic damage was evaluated at 24, 48, and 72 hpi. Morphological changes on the cell surface of *Mhp*-inoculated ALI-PRECs are indicated by black arrows. Scale bar: 100 μm.

Mock-inoculated ALI-PRECs showed no morphological changes (Fig. 1A). In contrast, cell morphology in ALI-PRECs was significantly altered following 2 and 5 h exposure to varying doses of *Mhp* (10^5.84^, 10^6.84^, and 10^7.84^ color-changing unit [CCU]/mL) (Fig. 1, *Mhp* inoculated). Both at 2 and 5 h incubation, pronounced cytopathic damage, such as compromised integrity of the pseudostratified epithelium with cell rounding and aggregation, was noticed as early as 24 h post-inoculation (hpi), particularly at higher doses of 10^6.84^ (Fig. 1C) and 10^7.84^ CCU/mL (Fig. 1D). At a dose of 10^5.84^ CCU/mL, these changes became more evident by 48 hpi with 5-h incubation and by 72 hpi (Fig. 1B) with 2-h incubation. At a dose of 10^7.84^ CCU/mL, morphological changes persisted through the latest time point irrespective of the time of exposure (Video S1). Despite these cellular alterations, ciliary motility persisted in *Mhp*-infected ALI-PRECs across all doses, time points, and exposure time durations (Video S2). Immunofluorescence assay (IFA) analysis revealed that these cellular alterations were *Mhp* specific as evidenced by the detection of *Mhp*-P46 in *Mhp*-infected ALI-PRECs at various doses used (Fig. S1).

### *Mhp* dose- and time point-dependent loss of ALI-PREC integrity

**Fig 2:**
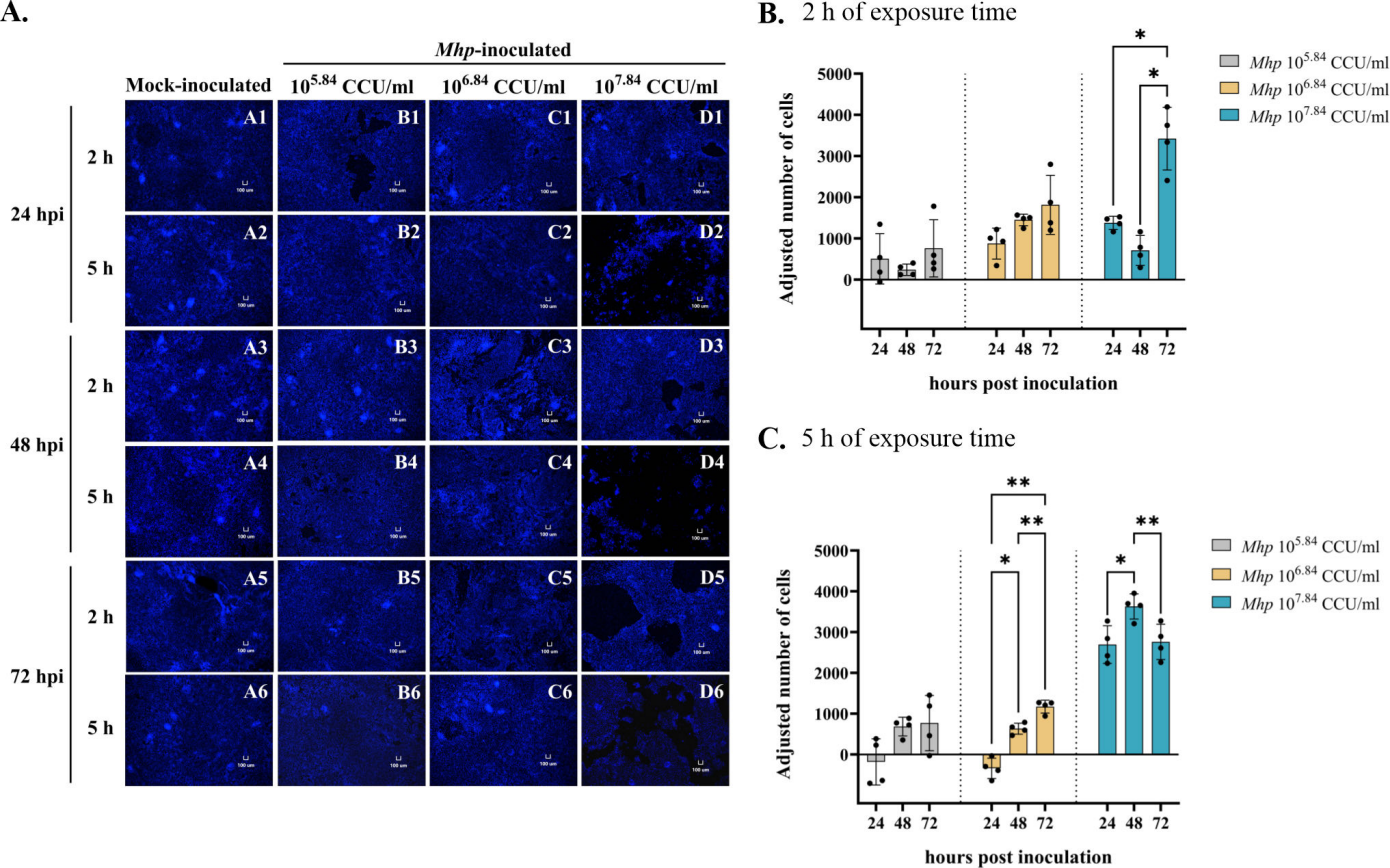
Dose- and time point-dependent loss of integrity in *Mhp*-inoculated ALI-PRECs. The integrity of *Mhp*-inoculated ALI-PRECs was assessed microscopically using IFA with DAPI cell nuclear staining. Infection medium served as the mock control. (**A**) Representative images at 40× magnification show a pronounced loss of integrity following 2 and 5 h of exposure to 10^7.84^ CCU/mL of *Mhp*. Scale bar: 100 µm. (**B and C**) Quantitative analysis of cell nuclei from micrographs captured at 100× magnification using Fiji Image J v2.14.0/1.54f software (NIH). Results for 2 and 5 h of exposure are shown in panels **B** and **C**, respectively. The number of nuclei was normalized to the corresponding mock-inoculated ALI-PRECs. Statistical analyses were performed using GraphPad Prism v10.2.1 (GraphPad, San Diego, CA, USA). *, *P* < 0.050; **, *P* < 0.005.

To further confirm the loss of epithelial cell integrity in *Mhp*-inoculated ALI-PRECs, 4′,6-diamidino-2-phenylindole (DAPI) nuclear staining, microscope assessment, and quantitative polymerase chain reaction (qPCR) were used to directly correlate the cell loss with reduced cell nuclei counts at 24, 48, and 72 hpi, compared to mock-inoculated cultures, as well as with the translocation of bacteria to the basolateral fraction. Mock-inoculated ALI-PRECs preserved epithelial continuity across all time points with 2 or 5 h incubation in contrast to *Mhp*-inoculated ALI-PRECs (Fig. 2A). ImageJ analysis and normalization of microscopic images showed significant loss of cells in ALI-PRECs infected with a high dose of 10^7.84^ CCU/mL of *Mhp* (Fig. 2A and D), with more evident effects after 5 h of exposure (Fig. 2C) compared to 2 h (Fig. 2B). Statistical analysis demonstrated sustained and significant differences in the adjusted cell numbers across dose (ANOVA, *P* = 0.002) (Fig. S2, left) and time points (ANOVA, *P* = 0.020) (Fig. S2, center). Although no significant difference in the adjusted number of cells among exposure time was detected (Fig. S2, right), exposure time’s interaction with dose and time point demonstrated a significant effect on the adjusted cell numbers (ANOVA, both *P* < 0.001) (Fig. 2B and C).

### Kinetics of *Mhp* growth and epithelial disruption in ALI-PRECs

**Fig 3:**
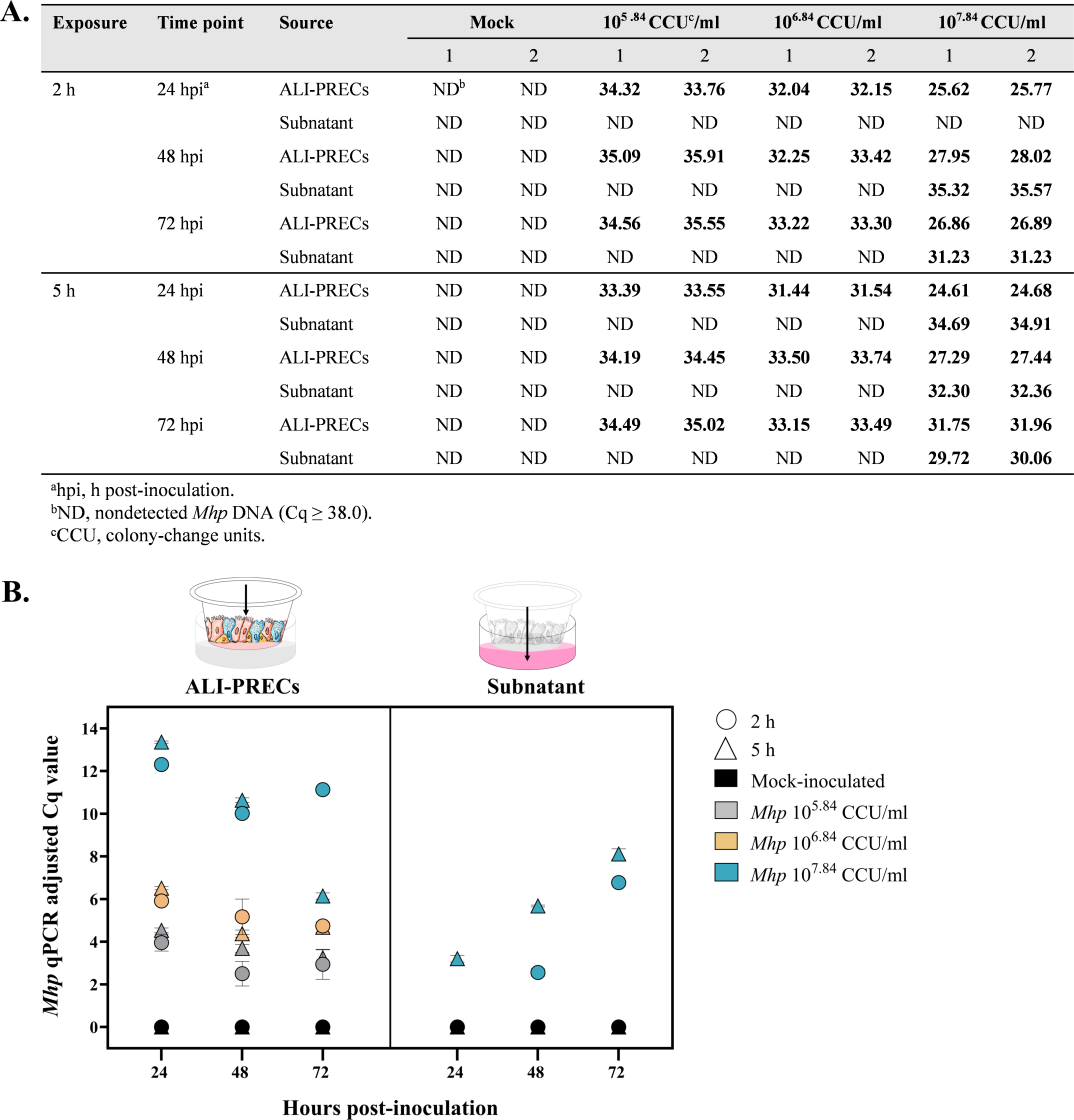
Dose-, time point-, and exposure time-dependent disruption of ALI-PRECs and basolateral migration of *Mhp*. (**A**) Cq values obtained from *Mhp* qPCR analysis of cell (ALI-PRECs) and basolateral (subnatant) fractions at specific time points (24, 48, and 72 hpi). (**B**) *Mhp* DNA was consistently detected in all *Mhp*-inoculated ALI-PRECs across time points. An increasing trend in adjusted Cq values (38 − sample Cq) was observed in the subnatant of ALI-PRECs inoculated with 10^7.84^ CCU/mL of *Mhp*, beginning at 24 hpi following 5 h of exposure and at 48 hpi following 2 h of exposure. Graphs were generated using GraphPad Prism v10.2.1 (GraphPad, San Diego, CA, USA).

*Mhp* growth kinetics were evaluated using qPCR. *Mhp* DNA was detected in both the cellular and subnatant fractions of inoculated ALI-PRECs (Fig. 3A). In the ALI-PREC cell fraction, a dose-dependent relationship was observed, with higher doses showing increased adjusted quantification cycle (Cq) values at 24 hpi, consistently evident across all time points, regardless of the incubation period (2 or 5 h) (Fig. 3B, ALI-PRECs).

Subnatant analysis revealed that *Mhp* DNA was undetectable at lower doses (10^6.84^ or 10^5.84^ CCU/mL) but detectable at 10^7.84^ CCU/mL (Fig. 3B, Subnatant). At 10^7.84^ CCU/mL, *Mhp* was detected at 48 and 72 hpi after 2 h of exposure but was detected as early as 24 hpi after 5 h of exposure, with adjusted Cq values increasing over time (Fig. 3B, Subnatant).

### Biological variability in cytopathic responses and *Mhp* persistence in ALI-PREC

To identify potential biological differences among individuals, we used a fixed dose of 10^7.84^ CCU/mL and 2 h incubation, and epithelial cell integrity was measured by microscopic evaluation and modulation of structural genes over time, recorded at every 24 h intervals up to 144 hpi.

**Fig 4:**
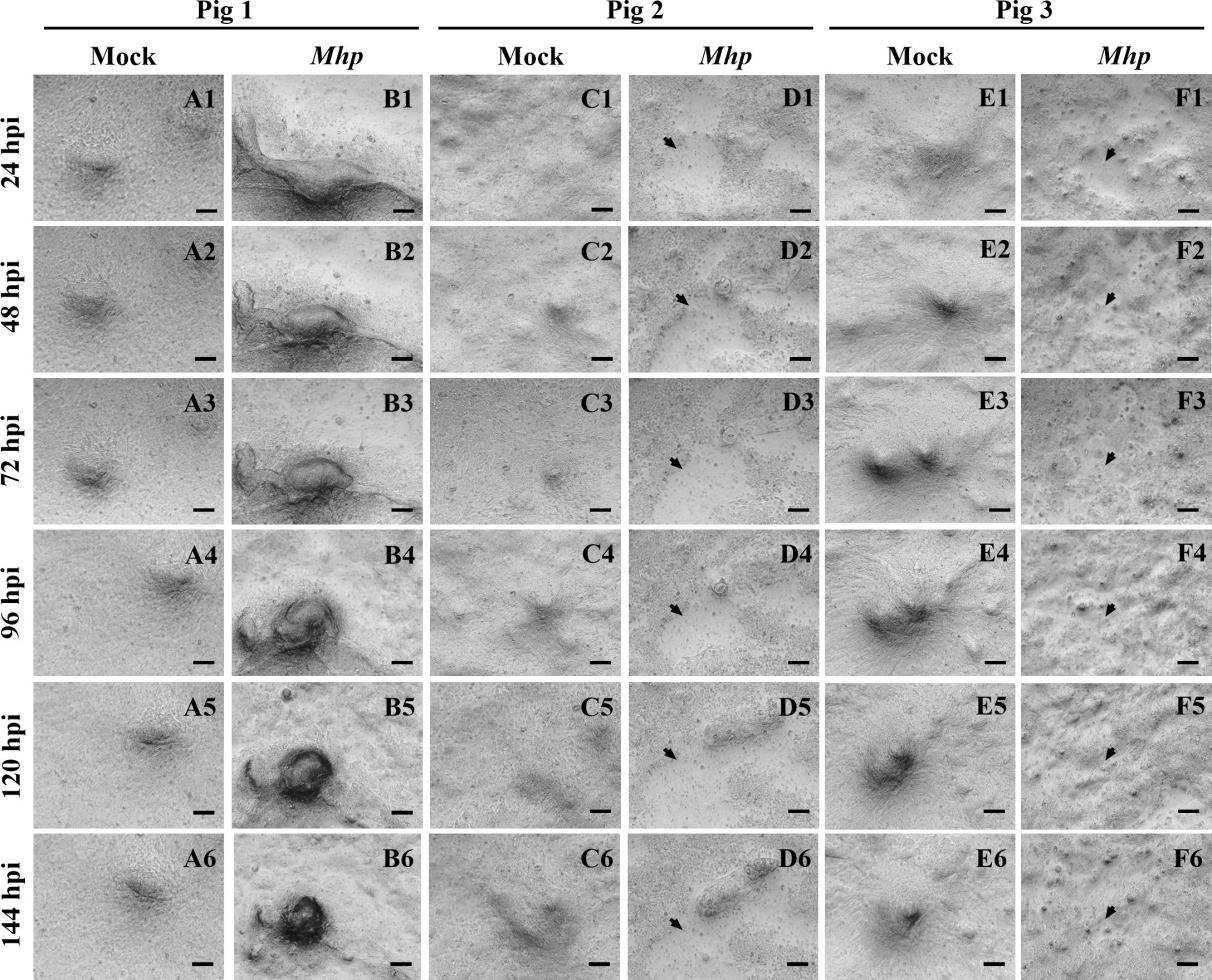
Temporal assessment of cytopathic damage in ALI-PRECs over time. ALI-PRECs were mock-inoculated with infection medium or exposed *Mhp*-inoculated to 10^7.84^ CCU/mL of *Mhp*. Following 2 h of exposure, cytopathic damage was assessed in the same field at 24, 48, 72, 96, 120, and 144 hpi. At 24 hpi, morphological changes such as cell aggregation, cell rounding, folding, and areas of discontinuity were observed in *Mhp*-inoculated ALI-PRECs. Black arrows indicate cell surface alterations. Scale bar: 100 μm.

Mock-inoculated ALI-PRECs derived from three independent pigs showed no morphological changes (Fig. 4A, C, and E). In contrast, *Mhp*-inoculated ALI-PRECs exhibited cytopathic damage, including cell aggregation, rounding, epithelial discontinuity, cytoplasmic stranding, and epithelium folding as early as 24 hpi, with increasing severity over time in two out of three pigs (Fig. 4B and D). Interestingly, while morphological changes were observed at 24 hpi in *Mhp*-inoculated ALI-PRECs from pig 3, epithelial discontinuity diminished after 96 hpi (Fig. 4F) (Video S3). Ciliary motility ceased at 24 hpi in pig 1, persisted up to 144 hpi in one technical replicate from pig 2, and remained present across all time points in all technical replicates (three per time point) from pig 3 (Video S2).

Normalized cell counts from DAPI nuclear-stained images revealed a significant difference in the cell numbers among *Mhp*-inoculated ALI-PRECs from different pigs (ANOVA, *P* = 0.008) (Fig. S3, left), but no differences were detected across time points (ANOVA, *P* = 0.256) (Fig. S3, right). The interaction between pig and time point had a significant effect on the cell numbers among *Mhp*-inoculated ALI-PRECs (ANOVA, *P* < 0.001) (Fig. S3, right). Data from each pig indicated a more pronounced decrease in cell numbers in ALI-PRECs derived from pig 1 and pig 2 than cells derived from pig 3 (Fig. S3).

**Fig 5:**
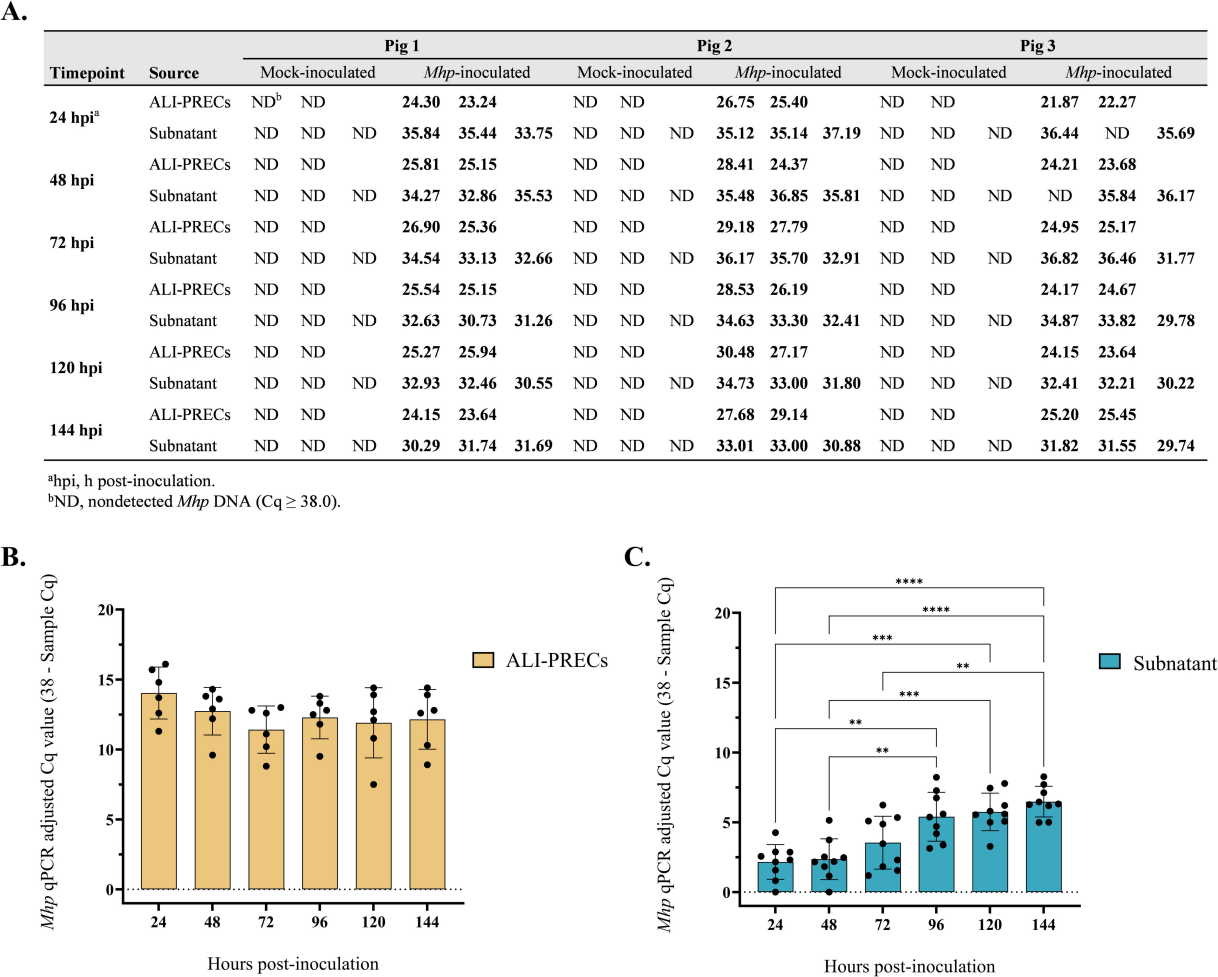
Disruption of ALI-PREC integrity and *Mhp* translocation over time. (**A**) Table showing Cq values from *Mhp* qPCR analysis of cells (ALI-PRECs) and basolateral (subnatant) fractions. (**B and C**) Graphs displaying adjusted Cq values (38 − sample Cq) from *Mhp* qPCR analysis of ALI-PRECs and basolateral fractions across 144 hpi. Statistical analysis and graph generation were performed using GraphPad Prism v10.2.1 (GraphPad, San Diego, CA, USA). **, *P* < 0.005; ***, *P* < 0.001; ****, *P* < 0.0001.

Further qPCR analysis demonstrated that *Mhp* DNA was undetectable in mock-inoculated ALI-PRECs across both cell and subnatant fractions over time (Fig. 5A, mock inoculated). In *Mhp*-inoculated ALI-PREC, qPCR revealed sustained higher adjusted Cq values in the cell fraction across all time points, maintaining these levels up to 144 hpi in all three pigs (Fig. 5B). In contrast, the subnatant fraction exhibited lower adjusted Cq values (up to 4.25) at 24 hpi, increasing significantly to 8.26 by 144 hpi (Tukey’s test, *P* < 0.001) (Fig. 5C).

**Fig 6:**
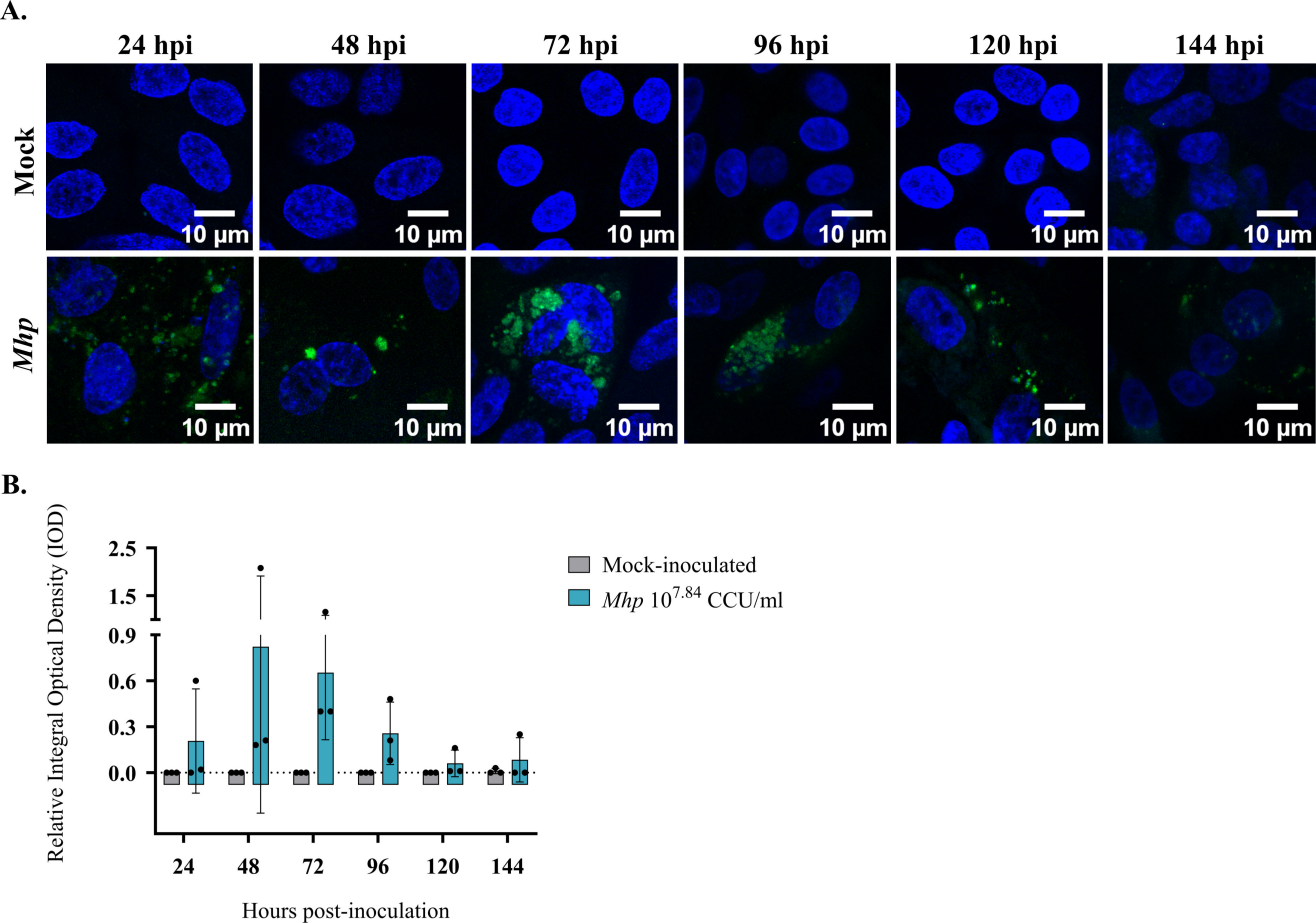
*Mhp* adheres to ALI-PRECs within 2 h of exposure at 10^7.84^ CCU/mL. (**A**) ALI-PRECs were mock-inoculated or exposed to *Mhp* 232 strain at a dose of 10^7.84^ CCU/mL. Following 2 h of exposure, the inoculum was removed; transwells were washed; and cell cultures were incubated at 37°C, 5% CO_2_, until fixation with 4% paraformaldehyde at 24, 48, 72, 96, 120, 144 hpi. IFA was performed to detect *Mhp*-P46 membrane protein (FITC) and cell nuclei (DAPI). Representative images show P46 detection over time in *Mhp*-inoculated ALI-PRECs. (**B**) The relative integral optical density (IOD) of FITC fluorescence was calculated using Fiji ImageJ v2.14.0/1.54f (NIH) by normalizing fluorescence intensity and area. Statistical analyses and graph generation were performed using GraphPad Prism v10.2.1 (GraphPad, San Diego, CA, USA). Data represent three independent cultures.

Confocal imaging of identifying *Mhp*-P46 membrane protein via IFA provided further evidence of bacteria-associated cytopathic changes in ALI-PRECs (Fig. 6). The levels of FITC-labeled *Mhp*-P46 were quantified using relative integrated optical density (IOD) measurements which directly correlate with fluorescence intensity. *Mhp*-P46 fluorescence was consistently detected until 120 hpi across all biological replicates (Fig. 6A), peaking at 48 hpi and declining steadily by 144 hpi (Fig. 6B) in all three pigs. Microscopic assessment and IFA also revealed that areas of epithelium dislodgment were indeed strongly positive for *Mhp*-P46 in *Mhp*-inoculated ALI-PRECs (Video S4).

### *Mhp* modulates the expression of genes related to adherens junctions and ciliary motility

**Fig 7:**
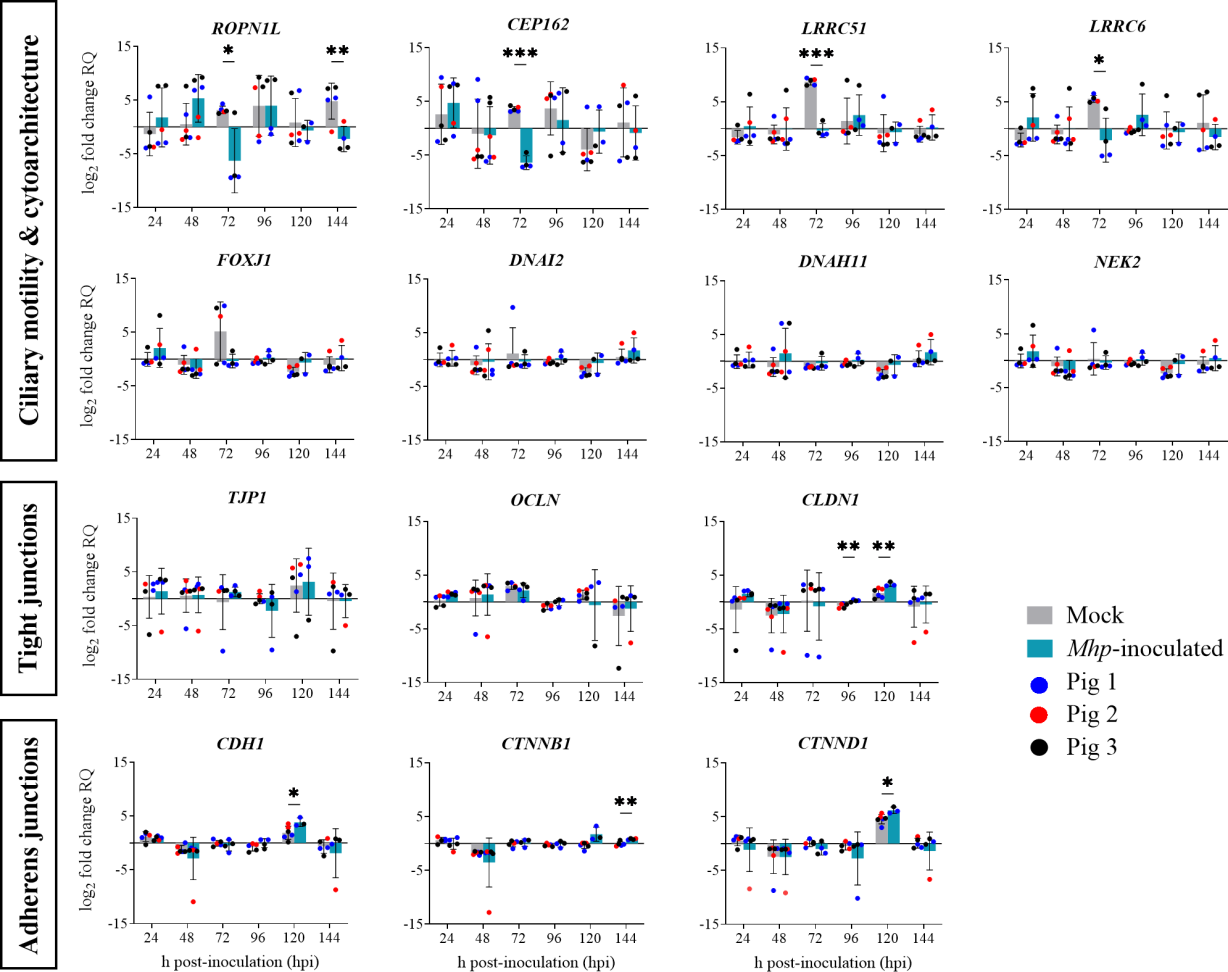
Transcriptional regulation of genes related to cytoarchitecture, ciliary motility, and intercellular junctions in ALI-PRECs inoculated with *Mhp*. Bar graph showing log_2_ fold change relative quantification (RQ) levels of 14 porcine genes (*ROPN1L*, *CEP162*, *LRRC51*, *LRRC6*, *FOXJ1, DNAI2, DNAH11, NEK2*, *TJP1*, *OCLN*, *CLDN1*, *CDH1*, *CTNNB1*, and *CTNND1*) measured in ALI-PRECs inoculated with 10^7.84^ CCU/mL of *Mhp* or mock-inoculated with culture medium at respective hpi (*x* axes). RQ values were calculated using the 2^−DD^*^CT^* method and normalized against the geometric mean of three endogenous control genes (*RPL10*, *GADPH*, and *PPIA*). Data represent three technical and three biological replicates. Statistical analyses and graphs generation were performed using GraphPad Prism v10.2.1 (GraphPad, San Diego, CA, USA). *, *P* < 0.050; **, *P* < 0.005; ***, *P* < 0.001.

*Mhp* significantly downregulated the expression of ciliary motility-related genes (*ROPN1L*, *LRRC51*, and *CEP162*) and cytoarchitecture-related genes (*LRRC6*) in *Mhp*-inoculated ALI-PRECs at 72 hpi (Tukey’s test, *p* < 0.05) (Fig. 7; ciliary motility and cytoarchitecture). Tight junction-related gene *CLDN1* was significantly upregulated at 96 and 120 hpi (Tukey’s test, *P* = 0.005), while *TJP1* and *OCLN* were not significant (Fig. 7, tight junctions). Adherens junction genes *CDH1* and *CTNND1* were upregulated at 120 hpi (Tukey’s test, *P* < 0.050), while *CTNNB1* was upregulated at 144 hpi (Tukey’s test, *P* = 0.007) (Fig. 7; adherens junctions).

## DISCUSSION

The ALI-PREC model has emerged as a robust platform for investigating host-bacterial interactions in respiratory diseases (12, 17, 18). Its capacity to mimic the complexity of porcine airway epithelium within an *in vivo*-like microphysiological environment makes it particularly suitable for studying pathogens such as *Mhp* (12, 19). Advances in ALI-PREC culture methodologies, including the incorporation of growth factors and refinements in transwell membrane properties, have significantly enhanced the model’s physiological relevance (15). These improvements have enabled comprehensive studies of airway-specific pathogens like *Mhp*, which predominantly target ciliated cells (19).

The susceptibility of tracheobronchial ALI-PRECs to *Mhp* strain 232, a widely used reference in the USA (20), was investigated to elucidate dose- and time point-dependent cytopathic effects (CPEs) and mechanisms underlying epithelial disruption. These findings reinforce the utility of ALI-PREC models in advancing knowledge of *Mhp* pathogenesis and guiding targeted interventions for swine health.

### Rapid adherence of *Mhp* to ALI-PRECs and dose dependency

A 2-min to 6-h adherence window was reported for *M. pneumoniae* in human bronchial-derived ALI-RECs (21, 22). Previous studies have shown that *Mhp* strain 91-3 attachment occurred within 90 min of exposure leading to epithelial damage (19). Even in the current study, rapid adherence of *Mhp* strain 232 to ALI-PRECs within 2 h at a dose of 10^7.84^ CCU/mL aligns with earlier reports, further corroborating the rapid adherence capability of mycoplasmas.

In contrast, Wang et al. proposed a 16 h exposure period for *Mhp* adherence in porcine bronchial-derived ALI-RECs, citing an undisclosed adhesion kinetic study and drawing parallels to findings obtained using PK-15 monolayers (12, 23). However, extrapolations between these models should be interpreted cautiously due to the inherent physiological and structural differences between primary cells and cell lines (24).

Interestingly, *Mhp* adherence appears dose dependent, with increased adherence correlating with higher bacterial loads, consistent with previous *in vitro* findings (25). However, factors such as strain heterogeneity, passage history, and culture conditions likely influence adhesion kinetics, necessitating cautious interpretation and generalization across *Mhp* strains (25).

### Epithelial disruption and cytopathic effects of *Mhp*

The capacity of *Mhp* to induce ciliostasis, loss of cilia, and robust inflammatory responses in the tracheal mucosa facilitates its evasion of mucociliary clearance and establishment of infection (1). Although previous studies have characterized *Mhp*-induced damage in ALI-PRECs using electron microscopy (12, 19), temporal tracking of cytopathic changes has been limited. This study leveraged polyethylene terephthalate transwells, enabling real-time microscopic assessment of cytopathic effects, a feature unavailable in earlier studies (19). This qualitative approach provides visual indicators of cytotoxicity, supporting the temporal assessment of *Mhp*-induced epithelial damage.

Cytopathic changes, including cell rounding, aggregation, cytoplasmic stranding, and loss of epithelial integrity, were dose and time point dependent, with significant disruption observed at 72 hpi to *Mhp* 10^7.84^ CCU/mL. These findings were consistent with previous *in vitro* studies that showed dose-dependent (5) and time point-dependent (26) cytopathic effects of *Mhp*. Epithelial disruption became apparent at 24 hpi but was delayed at lower doses (10^5.84^ and 10^6.84^ CCU/mL), corroborating earlier findings on the inoculum-dependent nature of cytopathic effects (5).

This study further demonstrated the loss of epithelium on transwells by DAPI nuclear staining and quantification of cell nuclei using ImageJ analysis. The use of transparent inserts with DAPI-stained cells has previously been shown to be an effective method for quantifying cell numbers on the membrane surface (27). This technique overcomes the limitations of intercalating dyes, which often generate high background fluorescence in ALI systems, particularly when membrane integrity is compromised or when variations in cell phenotype, morphology, and yield impede uniform single-cell dissociation.

Cell loss was directly correlated to decreased nuclear count, suggesting compromised epithelial integrity. The discontinuity in ALI-PRECs with dislodged epithelial cells was frequently marked by *Mhp*-P46. This finding aligns consistently with observations from prior studies (10, 12). The dose-dependent nature of epithelial disruption was further confirmed, with bacterial detection in the basolateral chamber restricted to higher inoculum doses (10^7.84^ CCU/mL), consistent with earlier findings emphasizing the impact of bacterial load on epithelial damage (5).

Variations in susceptibility to *Mhp* between tracheal- and lung-derived epithelial cells have been previously documented *in vivo* (10), attributed to differences in cell populations, diversity, proportions, and ultrastructural constitution across airway segments (28). These intrinsic differences must be considered when comparing models from different REC sources.

Although not the primary focus of this study, well-established methods, such as biochemical assays, gene expression analyses, and protein profiling, could elucidate the mechanisms behind the observed decrease in cell nuclei. Additionally, it remains unclear whether the morphological changes result directly from bacteria contact or from their secreted metabolic byproducts.

### Biological variability in cytopathic responses and *Mhp* persistence

The observed variability in cytopathic responses among pigs highlights significant interindividual differences in susceptibility to *Mhp* infection. While all pigs exhibited cytopathic damage, the extent of epithelial disruption varied, with one pig showing reduced epithelial discontinuity after 96 hpi. Additionally, ciliary motility persisted up to 144 hpi in one technical replicate and remained consistent in another pig throughout all time points.

These variations suggest that host-specific factors, such as genetic differences, innate immune responses, and inherent epithelial cell characteristics, critically influence the severity and progression of *Mhp*-induced damage. Additionally, strain-dependent differences in *Mhp* virulence (29) and breed-associated host susceptibility (30) have been shown to influence clinical outcomes, reflecting the complexity of host-pathogen interactions. Similar interindividual variability has been documented in other infectious models, where genetic and immunological diversity among hosts leads to varied disease outcomes (31, 32). Normalized cell counts from DAPI nuclear-stained images revealed significant differences in cell damage based on the animal, suggesting innate interanimal variability, which could be a genetically determined trait that confers greater resilience against *Mhp* infection. Understanding the mechanisms driving this variability is essential for developing targeted interventions and improving disease management strategies. It will be interesting to characterize genomic and proteomic biomarkers of resistance or susceptibility to *Mhp* and investigate specific immune responses and epithelial repair mechanisms, which were beyond the scope of the present study.

### Transcriptional modulation by *Mhp* and repair mechanisms

Epithelial cells are connected by tight and adherens junctions, which regulate transepithelial transport and maintain epithelial integrity. These structures are commonly targeted by respiratory pathogens to disrupt the epithelium, as was also demonstrated in ALI-RECs (12, 33, 34). A previous study using porcine bronchial-derived ALI-RECs reported a significant reduction in ZO-1 IOD during the first 28 hpi with *Mhp* (12). While informative, our study builds on these findings by examining the transcriptional regulation of both tight and adherens junction genes and extending the observation window to 144 hpi. In this study, *Mhp* modulated the expression of genes related to ciliary motility and cytoarchitecture (*ROPN1L*, *LRRC51*, *CEP162*, and *LRRC6*), tight junction-related genes (*CLDN1*), and adherens junctions (*CDH1*, *CTNNB1*, and *CTNND1*) in ALI-PRECs, suggesting its role in the regulation of epithelial barrier integrity and ciliary motility. The downregulation of *LRRC6* observed at 72 hpi likely impairs basal body organization and ciliogenesis, contributing to the observed decrease in ciliary beating. *LRRC6* facilitates the nuclear localization of *FOXJ1*, a critical transcription factor required for motile cilia formation; conversely, absence of *LRRC6* has been shown to hinder the assembly of dynein arm protein complexes that are essential for ciliary beating (35). In parallel, the downregulation of centriole-related gene *CEP162*, which performs a critical role in ciliogenesis, suggests a coordinated response affecting ciliary and epithelial architecture (36). Despite this downregulation, no changes were observed in *FOXJ1*, dynein-related (*DNAH11* and *DNAI2*), and centriole-related gene *NEK2*, highlighting the selective transcriptional impact of *Mhp* on components of the ciliary program.

Adherens junctions, composed of E-cadherin (encoded by *CDH1*) and catenins (encoded by *CTNNB1* and *CTNND1*), form interfaces with the microtubule network and actin cytoskeleton, playing essential roles in cell proliferation, differentiation, and epithelial restoration (37). Studies in tracheal organ cultures and ALI-PRECs on influenza A virus have shown that basal cells contribute to epithelial repair by replacing damaged cilia (5, 38). Excessive activation of E-cadherin has been linked to abnormal goblet cell proliferation, potentially influencing airway epithelial responses (39). It is tempting to speculate that goblet and basal cells may be involved in the repair process observed in ALI-PRECs, as shown previously in other humans and murine ALI models (40, 41).

The downregulation of *LRRC51* and *ROPN1L*, essential for coordinated ciliary beating, further supports the hypothesis that *Mhp* impairs ciliary motility through transcriptional modulation. Although mutations in these genes do not affect cilia formation, their loss significantly reduces ciliary beat frequency (42). This downregulation may underline the sustained ciliostasis observed in the ALI-PRECs from one biological replicate. Notably, ciliary beating persisted until 144 hpi in two biological replicates, underscoring interindividual variability in response to *Mhp* infection. These transcriptional changes demonstrate the ability of *Mhp* to modulate host gene expression to facilitate its survival and pathogenicity, aligning with findings from other respiratory pathogens (43).

Moreover, the persistence of ciliary motility in some biological replicates suggests that certain host factors may enhance recovery or maintenance of ciliary function despite infection, potentially through robust epithelial repair, differential gene expression, or protective proteins. Although ciliary motility was assessed qualitatively (absence or presence), results were consistent within biological replicates yet varied across treatments, suggesting differences in beat frequency. Quantitative analysis could determine whether these differences are significant and correlated with individual responses to the pathogen.

These findings emphasize the importance of considering host-specific factors in experimental designs and interpretations, enabling a better understanding of host responses and informing the development of more effective, directed treatment strategies.

### Limitations of the study

Despite the comprehensive findings, this study has some limitations. The use of a single *Mhp* strain may not capture the full spectrum of pathogenic variability among different strains. Validating these findings with complementary protein-level analysis would further strengthen the interpretation of the transcriptional changes observed in this study. Other approaches to evaluate epithelial integrity, including transepithelial resistance measurements and junction-specific immunostaining, were not performed and would have provided additional support for the present findings. Additionally, the ALI-PREC model, while robust, does not incorporate immune cells, which play a critical role in host-pathogen interactions *in vivo*. Furthermore, the study was limited to three biological replicates, which may not fully account for the observed interindividual variability. Future research should explore the effects of different *Mhp* strains on ALI-PRECs to understand the broader applicability of the findings. Investigating the role of immune cells in the ALI-PREC model could provide a more comprehensive understanding of host-pathogen interactions. Additionally, further mechanistic studies are needed to elucidate the specific pathways through which *Mhp* modulates gene expression and induces cytopathic effects.

### Conclusions

This study demonstrates that *Mhp* strain 232 adheres rapidly and dose-dependently to ALI-PRECs, inducing significant cytopathic damage and modulating gene expression related to ciliary motility and intercellular junctions. The observed host-specific variability in attachment and infection underscores the importance of using several biological replicates while performing *in vitro* studies. The ALI-PREC model is a suitable *in vitro* respiratory epithelium model to evaluate early *Mhp* interaction at a mucosal level, providing valuable insights into pathogenesis and identifying potential targets for therapeutic intervention.

## MATERIALS AND METHODS

### Experimental design

**Fig 8:**
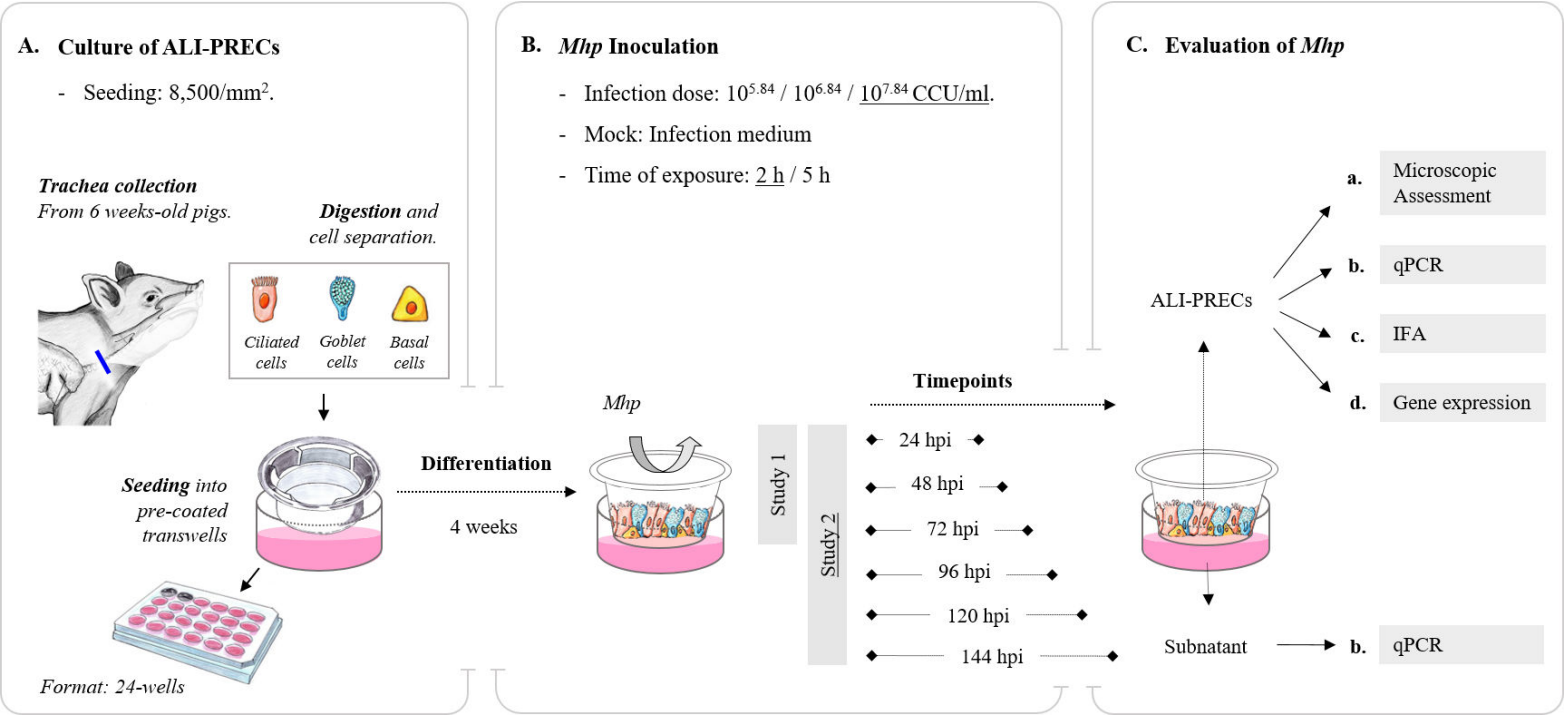
Experimental design for ALI-PREC culture and *Mhp* infection studies. (**A**) ALI-PREC culture: tracheas from 6-week-old pigs (*n* = 3) were dissected, washed, and enzymatically digested to isolate PRECs, which were seeded into pre-coated transwells in a 24-well format. PRECs were cultured under ALI conditions for 4 weeks to allow differentiation. (**B**) *Mhp* inoculation: for the susceptibility study, differentiated ALI-PRECs (day 30) were exposed to infection medium (mock inoculation) or inoculated with 10^5.84^, 10^6.84^, and 10^7.84^ CCU/mL *Mhp* strain 232. After 2 or 5 h at 37°C, 5% CO_2_, the inoculum was removed; cells were washed and incubated for 24, 48, and 72 hpi. For the kinetic study, ALI-PRECs were inoculated with 10^7.84^ CCU/mL of *Mhp* for 2 h, followed by incubation in fresh infection medium for up to 144 hpi. (**C**) Evaluation of infection: the infection outcome was assessed microscopically by IFA and by analyzing transcriptional regulation of structural genes. *Mhp* DNA detection was performed using qPCR on subnatants collected from the basolateral compartment and on cell lysates.

To determine the susceptibility of ALI-PRECs to *Mhp* infection, we employed a dose-response approach. Three doses of *Mhp* (10^5.84^, 10^6.84^, and 10^7.84^ CCU/mL), along with mock control (infection medium), were inoculated on the ALI-PRECs and incubated for either 2 or 5 h at 37°C with 5% CO_2_ to facilitate bacterial absorption. Following this incubation, the integrity of the ALI-PRECs was assessed over the course of 24, 48, and 72 hpi by recording cell morphology and ciliary movement under a microscope. *Mhp* DNA and *Mhp* P46 antigen were evaluated using qPCR and IFA, respectively. After determining the susceptibility of ALI-PRECs to *Mhp*, *Mhp* infection kinetics (24, 48, 72, 96, 120, and 144 hpi) were evaluated by fixing a dose of 10^7^ CCU/mL and 2 h incubation. Next, the modulation of various genes associated with ciliogenesis, cytoarchitecture, and intercellular junctions (*ROPN1L*, *DNAH11*, *LRRC51*, *FOXJ1*, *DNAI2*, *NEK2*, *CEP162*, *LRRC6*, *TJP1*, *OCLN*, *CLDN1*, *CDH1*, *CTNNB1*, and *CTNND1*) was evaluated via qPCR analysis at each time point (Fig. 8).

### Trachea tissue processing and isolation of PRECs

The pigs from which tissues were collected were supplied by Struve Labs International, and the potential presence of other respiratory pathogens, including *Mycoplasma flocculare* and *Mycoplasma hyorhini*s, was ruled out prior to tissue collection. Prior to culture initiation, aliquots of tissue homogenate, cell culture medium, and the *Mhp* inoculum were submitted to the Iowa State University (ISU) Veterinary Diagnostic Laboratory and tested PCR negative for *M. hyorhinis* and *M. flocculare*. Aseptic collection of porcine tracheal sections (from below the larynx to the bronchial bifurcation) was performed on three 6-week-old healthy cesarean-derived, colostrum-deprived pigs. The sections were immediately transferred to a tube containing 50 mL of cold transport medium: Dulbecco’s minimum essential/Ham’s F-12 medium (DMEM/F12) with GlutaMAX (Thermo Fisher Scientific, Grand Island, NY, USA), supplemented with 100 IU/mL penicillin, 100 mg/mL streptomycin (Pen-Strep, Thermo Fisher Scientific), and 1.25 mg/mL amphotericin B (Thermo Fisher Scientific). The samples were transported to the laboratory on ice for further processing.

Isolation of PRECs was conducted as previously described (44). The isolated PRECs were counted and cryopreserved in 60% LHC basal medium (Thermo Fisher Scientific), 30% heat-inactivated EqualFETAL bovine serum (eFBS; EqualFETAL; Atlas Biologicals, Fort Collins, CO, USA), and 10% dimethyl sulfoxide (Millipore Sigma, St Louis, MO, USA) in liquid nitrogen (Fig. 8A).

### ALI-PRECs culture in transwell inserts

Transwell inserts (24-well; Sarstedt, Nümbrecht, Germany) were coated with type IV collagen from the human placenta (Millipore Sigma) and incubated at 37°C for 2 days. PRECs were thawed and resuspended in growth medium #1, which consisted of DMEM/F12 with GlutaMAX, supplemented with 5% heat-inactivated eFBS (Atlas Biologicals), 1× MEM non-essential amino acids (Millipore Sigma), 100 IU/mL of penicillin, 100 mg/mL of streptomycin (Thermo Fisher Scientific), 1.25 mg/mL of amphotericin B, and 1× HEPES (Thermo Fisher Scientific).

Each well of the plate was inoculated with 500 µL of growth medium #1, and each transwell insert was seeded with 8,500 cells/mm^2^. The plate was incubated at 37°C with 5% CO_2_. After 24 h, the growth medium #1 was replaced with growth medium #2, which included DMEM/F12 with GlutaMAX supplemented with 1,400 nM hydrocortisone (Acros Organics, Geel, Belgium), 2,700 nM L(-)-epinephrine (Acros Organics), 100 nM all-trans-retinoic acid (Acros Organics), 9.7 nM 3,3′,5-triiodo-L-thyronine (Cayman Chemicals, Ann Arbor, MI, USA), 0.5 ng/mL murine epidermal growth factor (PeproTech, Cranbury, NJ, USA), 1× insulin-transferrin-selenium-ethanolamine (Thermo Fisher Scientific), 1× HEPES, 2% Ultroser-G (Sartorius, Cergy-Saint-Christophe, France), 100 IU/mL of penicillin, 100 mg/mL of streptomycin (Thermo Fisher Scientific), and 1.25 mg/mL of amphotericin B. The cells were then incubated again at 37°C with 5% CO_2_.

Growth medium #2 was replaced every 2–3 days until the differentiation of PRECs into ALI-PRECs. After 15 days, microscopic evaluation using an inverted microscope (Olympus CKX4; Olympus, Center Valley, PA, USA) revealed the presence of mucus and ciliary movement. The differentiation continued through days 28–30. Once the ALI-PRECs were fully differentiated, they were used for *Mhp* infection studies.

### *M. hyopneumoniae* inoculation into ALI-PRECs

Two days prior to inoculation, the ALI-PRECs were washed twice with 300 µL of LHC basal medium (Thermo Fisher Scientific) on both the apical and basal sides. The growth medium #2 in the plate wells was then replaced with infection medium, composed of DMEM/F12 with Glutamax supplemented with 2% Ultroser G, 1× MEM non-essential amino acids, 1× HEPES, 100 IU/mL of penicillin, and 100 mg/mL of streptomycin (Thermo Fisher Scientific).

*Mhp* pathogenic strain 232 (donated by Boehringer Ingelheim Animal Health USA Inc., passage 5) was propagated in Friis medium and prepared as single-use frozen aliquots at a titer of 10^7.84^ CCU/mL. Aliquots were stored at −80°C and thawed once immediately prior to use. Working 10-fold dilutions (10^6.85^ and 10^5.84^ CCU/mL) were prepared from the thawed aliquot immediately before inoculation to ensure targeted CCU administration. No frozen stock was subjected to repeated freeze-thaw cycles to minimize loss of viability.

The ALI-PRECs were inoculated with 300 µL of *Mhp* at doses of 10^5.84^, 10^6.85^, or 10^7.84^ CCU/mL, or mock-inoculated with infection medium or Friis medium as negative controls. PCR assays targeting *M. hyorhinis* and *M. flocculare* were performed to verify that neither bacteria were present in the stock inoculum or the cell culture medium. Friis medium is a liquid growth medium used for the isolation and laboratory adaptation of multiple *Mhp* strains, including the *Mhp* strain 232 utilized in this study (45, 46). The plate wells received 500 µL of infection medium and were incubated at 37°C with 5% CO_2_ for 2 or 5 h to allow bacterial adhesion to the cells (Fig. 8B). After inoculation, the inoculum was removed from the transwell inserts, and the cells and plate wells were rinsed once with 300 and 500 μL of LHC basal medium (Thermo Fisher Scientific), respectively. Fresh infection medium (1 mL) was then added to the plate wells, which were incubated at 37°C with 5% CO_2_.

At 24 hpi intervals (Fig. 8C), the ALI-PRECs were observed under an inverted microscope (Olympus BX 53/43) to assess CPE and ciliary movement. One transwell insert from each treatment group was fixed with 300 µL of 4% paraformaldehyde (PFA; Electron Microscopy Sciences, Hatfield, PA, USA) for 15 min at room temperature (RT; 24°C) to detect the presence of *Mhp* P46 protein. A second transwell insert from each treatment was inoculated with 100 µL of cold TRIzol (Thermo Fisher Scientific) to lyse the cells. After a 10-min incubation at RT, the cell lysates were transferred to 1.5 mL microcentrifuge tubes (Eppendorf LoBind microcentrifuge tubes; Eppendorf SE, Hamburg, Germany) and stored at −80°C for PCR analyses. Cells from a third transwell insert, corresponding to the *Mhp* infection kinetic experiment of this study, were lysed following the same procedure. The subnatant from the plate wells was collected in microcentrifuge tubes (Eppendorf SE) for subsequent detection of *Mhp* DNA via qPCR analysis. Subnatants of each treatment group were collected from two (Fig. 8B, susceptibility study) and three (Fig. 8B, kinetic study) individual transwells.

### Microscopic assessment

The presence or absence of cytopathic damage and ciliary motility was assessed microscopically over time in both *Mhp*- and mock-inoculated ALI-PRECs. Micrographs and videos were captured and processed using an Olympus BX 53/43 microscope equipped with an Olympus DP80 camera and Olympus cellSens Dimension 1.16 imaging software.

### *Mhp* DNA qPCR

DNA extractions were performed using the MagMAX Pathogen RNA/DNA extraction kit (Thermo Fisher Scientific) on a KingFisher Apex magnetic-particle processor (Thermo Fisher Scientific) following the manufacturer’s instructions. Two negative extraction controls (NECs) and two positive extraction controls (PECs) were included with each extraction run. An internal control (IC) supplied with the commercial Tetracore kit (EZ-M. Hyo Real-Time PCR; Tetracore Inc., Rockville, MD, USA) was added to the lysis buffer at the start of each extraction (6 μL per sample) to monitor extraction efficiency and PCR inhibition; successful amplification of the IC confirms extraction and amplification without inhibition. Eluted DNA was stored at −80^o^C until qPCR analysis.

*Mhp* detection was performed using the EZ-M. Hyo Real Time PCR kit (Tetracore Inc.) according to the manufacturer’s instructions. This commercial assay targets multiple proprietary regions of the *Mhp* genome; exact primer/probe sequences are not publicly available. Each qPCR run included the manufacturer’s positive and negative controls, the NECs and PECs described above, and no-template controls (NTCs). Reaction mixtures (final volume 25 μL) contained 17.25 μL *Mhp* qPCR master mix, 0.75 μL enzyme blend (DNase inhibitor), and 7 μL extracted DNA (containing the IC). To verify that PCR results reflected relative changes in bacterial load, a kit-specific standard curve was generated from 10-fold serial dilutions of *Mhp* inoculum (10⁷–10°CCU/mL) and run in parallel (data not shown).

The qPCR was conducted on an Applied Biosystem 7500 Real-Time PCR System (Thermo Fisher Scientific) using the following cycling conditions: 48°C for 15 min and 95°C for 2 min for holding, followed by 45 cycles of 95°C for 5 s for denaturation and 60°C for 40 s for amplification. Data analysis was performed using Sequence Detection Software v1.4 (Applied Biosystems). Samples with a threshold of Cq above 38 were considered negative. Data were presented as the mean ± standard deviation (SD) of the adjusted Cq values (38 − sample Cq).

### *Mhp* P46 membrane protein IFA

The PFA-fixed ALI-PRECs in transwell inserts were permeabilized with 50 μL of 0.1% Triton X-100 (Millipore Sigma) for 10 min at RT and subsequently washed twice with 300 µL of phosphate-buffered saline (PBS). The ALI-PRECs were incubated with 100 μL of mouse monoclonal anti-*Mhp*-P46 (D79.1-7, ISU Veterinary Diagnostic Laboratory), diluted 1:100 in PBS containing 0.1% bovine serum albumin (BSA; Jackson ImmunoResearch Inc., West Grove, PA, USA), for 1 h at 37°C. After three washes with 200 μL of PBS, 100 μL of FITC-conjugated goat antimouse IgG antibody (KPL; SeraCare Life Sciences, Milford, MA, USA), diluted in PBS with 0.1% BSA (20 μg/mL), was added to each transwell and incubated for an additional hour at 37°C.

After three more washes with 200 μL of PBS per well, cell nuclei were stained by adding 50 μL of NucBlue Fixed Cell Reagent (DAPI, Thermo Fisher Scientific), diluted in PBS with 0.1% BSA (two drops/mL), and incubating for 5 min at RT. The transwell inserts were then washed three times with 500 µL of PBS, and 100 μL of PBS was added to each well before imaging.

Images were captured using an inverted Olympus CKX4 microscope equipped with an Infinity 2 camera and Infinity Analyze v6.5.5 imaging software (Lumenera Corp, Ottawa, ON, Canada), as well as a Zeiss LSM 700 Laser Scanning Confocal Microscope (Carl Zeiss, Jena, Germany) using Zen Black imaging software v14.0.27.201 (Carl Zeiss). Microscope settings were normalized based on mock-inoculated fluorescence, and a well was classified as *Mhp*-P46-positive if specific staining was observed. For every IFA run and time point, three negative controls were processed in parallel: (i) no-primary antibody controls (secondary only), (ii) no-secondary-antibody control (primary only), and (iii) mock (non-inoculated) cultures. Microscope exposure and detector settings were set on mock controls and kept constant for paired image acquisition. Isotype-matched controls and antigen pre-absorption were not performed. Images were captured as single shots (Olympus CKX4) or as z-stacks (Zeiss LSM 700 CLSM).

Z-stack images were processed in split channels with automatic background subtraction. Clean z-stacks were then combined as hyperstacks to create z-composite maximum-intensity projection images or to produce videos. The IOD of *Mhp* P46 fluorescence staining in the ALI-PRECs was analyzed using Fiji ImageJ software v2.14.0/1.54f (developed by the National Institute of Health) (47). Each image was processed with the maximum entropy algorithm to adjust the threshold, and the OD values were generated as a function of the mean gray values. The integrated optical density was calculated based on total area, percentage of area, and mean gray values (calibrated to OD values). Data were presented as the mean ± SD of two microscopic fields per well across three independent ALI-PREC cultures derived from three different animals.

### DAPI cell nuclear staining for evaluating ALI-PREC integrity

PFA-fixed ALI-PRECs were counterstained with DAPI nuclear staining to demonstrate the cell integrity on transwell membranes. Micrographs of DAPI-stained nuclei were captured at 40× and 100× magnifications and processed using Fiji-ImageJ software. Quantification of cell nuclei was performed in four fields per image using symmetric grids at 100× magnification. Images were processed by splitting the RGB channel, with the blue channel selected for analysis to suppress the noise during particle counting. Background fluorescence was subtracted from each image by adjusting the particle size threshold settings to ≤255 to minimize overlapping nuclei and facilitate accurate particle recognition. Image segmentation and threshold adjustments were performed using the Watershed tool. Quantitative measurements included total particle count, area, average size, and the percentage of area covered by particles. The normalized number of nuclei was calculated by comparing the particle count in *Mhp*-inoculated ALI-PRECs to the corresponding values from mock-inoculated controls, yielding an adjusted cell count per treatment. Data were presented as the mean ± SD of the adjusted cell count derived from four microscopic fields per image.

### Gene expression

#### Total RNA extraction and cDNA synthesis

Total RNA was extracted from ALI-PRECs preserved in TRIzol using the E.Z.N.A. Total RNA Kit (Omega Bio-tek, Inc., Norcross, GA, USA) according to manufacturer’s instructions. RNA was eluted and quantified with a NanoDrop one microvolume UV-Vis spectrophotometer (Thermo Fisher Scientific). Samples with an A260/280 ratio between 1.42 and 2.05 were selected for further analysis. cDNA synthesis was performed using 20 ng of total RNA per sample with the qScript XLT cDNA SuperMix (QuantaBio, Beverly, MA, USA) following the manufacturer’s protocol. Synthesized cDNA was stored at −20°C until use.

#### Gene expression analysis

This study involved transcriptional analysis of 15 target genes related to ciliary motility, cytoarchitecture, and intercellular junctions, as well as seven endogenous controls (*ACTB*, *B2M*, *GADPH*, *PPIA*, *RPL10*, *EIF3K*, and *PCNA*) in ALI-PRECs (Table 1). All samples and reagents were dispensed to individual 384-well plates using an Automated Liquid Handling instrument (Bravo; Agilent Technologies, Santa Clara, CA, USA) to be used on SmartChip Real-time PCR system (Takara Bio USA, Inc., Mountain View, CA, USA). Each run of the SmartChip Real-time PCR system can perform 5,184 real-time PCR reactions with a volume of 200 nL each, and they were filled using the SmartChip Multisample Nanodispenser in 24 genes/216 samples format. Cells from each pig were seeded into two independent transwells for every treatment-time point combination, and each transwell was processed as an individual sample (*n* = 72). All qPCR reactions were performed using 1× PowerUp SYBR Green Master Mix (Thermo Fisher Scientific), 500 nM of swine-specific primers (Millipore Sigma) (Table 1), and 0.34 ng of total RNA converted to cDNA. The cycling conditions used were 50°C for 2 min and 95°C for 2 min holding; 40 cycles, 95°C for 15 s denaturation and 60°C for 1-min amplification. Final melting curve analysis was performed at 95°C for 15 s, 60°C for 1 min, and 95°C for 15 s. All qPCR reactions were performed in triplicate, and NTCs were included in each plate/chip.

**TABLE 1:** Primers used for gene expression analysis

Biological activity	Gene ID	Associated gene name	Description		Sequences (5′−3′)	Reference^*a*^
Ciliary motility	XM_021076812.1	ROPN1L	Rhophilin-associated tail protein 1 like	F	AATGCCCATGGCCACTCA	*
				R	TGGCTACACTGCTTGTGCAAA	*
	XM_021063431.1	DNAH11	Dynein axonemal heavy chain 11	F	ATGTGTGTCGCATCAGCAGAA	*
				R	GCCCCCCACGCCAAT	*
	NM_001282115.1	LRRC51	Leucine-rich repeat-containing protein 51	F	GGACACGTCGGTGCAAGAG	*
				R	TGGCTCCTCGCTTAACAGATC	*
	XM_003357959.4	FOXJ1	Forkhead box J1	F	GGATCACGGACAACTTCTGCTACT	*
				R	CGCGGCACCTTGATGAA	*
Cytoarchitecture	XM_005668635.3	DNAI2	Dynein axonemal intermediate chain 2	F	CAGCGAGTTCGGGAAGCA	*
				R	TGGGCTGGACTGTCGATGTT	*
	XM_003130413.3	NEK2	NIMA-related kinase 2	F	GGCCCTAAAGGAATGTCACAGA	*
				R	TTGCCATCCAGGAAAACGTT	*
	XM_005659266.3	CEP162	Centrosomal protein 162	F	TGAAGATGGGCCCACATGT	*
				R	TGCACCGTTGTTTCCTATACCTT	*
	NM_001243334.1	LRRC6	Leucine-rich repeat containing 6	F	GGTACATGGACACCTCTTTAATCGA	*
				R	GCACGAGCTGAAATGGCTTT	*
Intercellular junctions	XM_003480423	TJP1	Tight junction protein 1	F	GAGGAAACAGCTATATGGGAACAAC	*
				R	TTGACGTTTCCCCACTCTGAA	*
	NM_001163647.2	OCLN	Occludin	F	GAGCAGCCCCCCAATGTT	*
				R	TCTCCGCATAGTCCGAAAGG	*
	NM_001244539.1	CLDN1	Claudin 1	F	CCCCAGTCAATGCCAGATATG	*
				R	AAAGTAGGGCACCTCCCAGAA	*
	NM_001163060.1	CDH1	Cadherin 1	F	GAACCCACAGCCTCATGTCA	*
				R	CCCATGTGTTAGTTCTGCAGTGA	*
	NM_214367.1	CTNNB1	Catenin beta 1	F	GGCTCTTGTGCGTACTGTCCTT	*
				R	GGTGTCGGCTGGTCAGATG	*
	XM_005660889.3	CTNND1	Catenin delta 1	F	GCCTCCTTCGGAACTTGTCATAT	*
				R	ATTGGGAGGTGCCTCTTGGTA	*
Housekeeping genes	AY550069.1	ACTB	Cytoskeletal beta actin	F	CTCTTCCAGCCCTCCTTCCT	(16)
				R	CGACGTCGCACTTCATGATG	(16)
	NM_213978.1	B2M	Beta-2-microglobulin	F	CCCCCGAAGGTTCAGGTTTA	(16)
				R	GCAGTTCAGGTAATTTGGCTTTC	(16)
	NM_001206359.1	GADPH	Glyceraldehyde-3-phosphate dehydrogenase	F	CCCCAACGTGTCGGTTGT	(16)
				R	CCTGCTTCACCACCTTCTTGA	(16)
	NM_214353.1	PPIA	Peptidylprolyl isomerase	F	GGTCCTGGCATCTTGTCCAT	(16)
				R	TGGCAGTGCAAATGAAAAACTG	(16)
	NM_001044543.1	RPL10	Ribosomal protein L10	F	CCACATTGGCCAAGTCATCAT	(16)
				R	CTGCGCAGGGCCTCAA	(16)
	XM_005664595.3	EIF3K	Eukaryotic translation initiation factor 3 subunit K	F	ACCAGTTCAACCCAGCTTTCTTC	(16)
				R	GGTTGGTGAGGGCCTTCAG	(16)
	NM_001291925.1	PCNA	Proliferating cell nuclear antigen	F	TACGCTAAGGGCAGAAGATAATGC	(16)
				R	TGAGATCTCGGCATATACGTG	(16)

^*a*^ The asterisks indicate that they were designed for this study.

Finally, qPCR results were analyzed using SmartChip qPCR software v2.8.100.0 (Takara Bio USA, Inc.) and exported to Microsoft Excel. Amplification efficiencies beyond the range (1.5–2.2), samples with multiple melting peaks and above 40 Cq, were discarded. Cycle threshold values equivalent to Cq values were subsequently analyzed on qBase+ gene expression analysis software (Biogazelle, Zwijnaarde, Belgium), which calculates the stability of endogenous control genes and provides a value called *M* value. Out of seven endogenous control genes in both *Mhp* inoculated and mock inoculated, three genes (*RPL10*, *GADPH*, and *PPIA*) were identified as the best endogenous controls based on their low *M* value. Hence, gene expression data were normalized using the geometric mean of these three genes (48). Prior to statistical analysis, qPCR samples were evaluated for technical replicate performance and only retained samples if at least two of three technical replicates produced amplification with comparable Cq values. In accordance with MIQE guidelines, samples with undetectable endogenous reference gene expression were excluded because normalization of target-gene expression was not technically valid (49); this exclusion occurred primarily in ALI-PRECs with severe *Mhp*-induced epithelial loss. Relative quantification (RQ) was calculated using the ΔΔCt method (50), and RQ values were log_2_-transformed for analysis. Results were reported as mean ± SD of log_2_-transformed RQ (log_2_ fold change RQ) for three biological replicates. No post hoc statistical outlier removal was performed: values that satisfied the QC criteria above were retained, including biologically extreme values, because they were judged to reflect true biological variation rather than technical failure. Missing observations resulting from infection-induced epithelial loss were accommodated in the statistical models using mixed-effects analysis (restricted maximum likelihood [REML]).

### Data analysis

The adjusted cell counts (DAPI nuclear staining), IOD (*Mhp*-P46 detection) from IFA, adjusted Cq values from qPCR, and log_2_-transformed RQ values from gene expression analyses were processed and analyzed using GraphPad Prism v10.2.1 (GraphPad, San Diego, CA, USA) and R v4.3.3 (R Foundation for Statistical Computing, Vienna, Austria). Assumptions of normality and homogeneity of variance were inspected by Shapiro-Wilk and Levene tests, respectively. When necessary, data were transformed (see below) prior to analysis.

For the susceptibility study, adjusted cell counts (mock inoculated vs *Mhp* inoculated) were analyzed by two-way ANOVA to assess the effects of exposure time, dose, time point, and their interactions. Pairwise comparisons were performed with Tukey’s post hoc test for multiple comparisons, and *P* values were adjusted to control a family-wise error rate of 0.05.

For the kinetic study, adjusted cell counts (mock inoculated vs *Mhp* inoculated) were analyzed by two-way ANOVA to assess the effects of pig, time points, and their interactions, followed by Tukey’s post hoc test. The same ANOVA approach was applied to adjusted Cq values (ALI-PRECs: two transwells, triplicates; supernatants: three transwells, triplicates) and to relative IOD values.

Relative quantification values were log_2_-transformed prior to analysis to stabilize variance and approach normality. Because missing observations produced an unbalanced repeated-measures design, log_2_-RQ data were analyzed using a mixed-effect model fitted with REML with infection status (mock- and *Mhp*-infected), time point, and their interaction as fixed effects and animal (pig) as random effect; multiple comparisons were adjusted with Tukey’s post hoc test. The mixed-effect analyses were implemented in R. Missing observations caused by *Mhp*-induced epithelial loss were accommodated by the REML model. Statistical significance was set at a *P* value of <0.05 (two sided).

## SUPPLEMENTAL MATERIAL

Fig. S1 spectrum.01998-25-s0001.tif Dose- and time point-dependent loss of integrity in Mhp-inoculated ALI-PRECs.

Fig. S2 spectrum.01998-25-s0002.tif Dose- and time point-dependent loss of integrity in Mhp-inoculated ALI-PRECs.

Fig. S3 spectrum.01998-25-s0003.tif Biological variability in loss of integrity in Mhp-inoculated ALI-PRECs.

Supplemental material spectrum.01998-25-s0004.docx Supplemental figure and video legends.

Video S1 spectrum.01998-25-s0005.mp4 Morphological changes associated with cytopathic damage in mock-inoculated and Mhp-inoculated ALI-PRECs at 72 hpi.

Video S2 spectrum.01998-25-s0006.mp4 Temporal assessment of the ciliary movement in mock-inoculated and Mhp-inoculated ALI-PRECs over 144 hpi.

Video S3 spectrum.01998-25-s0007.mp4 Temporal assessment of the cytopathic effect in mock-inoculated and Mhp-inoculated ALI-PRECs over 144 hpi.

Video S4 spectrum.01998-25-s0008.mp4 Immunofluorescence (IFA) staining for Mhp-P46 detection and DAPI cell nuclear staining in Mhp-inoculated ALI-PRECs at 24 hpi.

ASM does not own the copyrights to Supplemental Material that may be linked to, or accessed through, an article. The authors have granted ASM a non-exclusive, world-wide license to publish the Supplemental Material files. Please contact the corresponding author directly for reuse.
