# Supplementary material for: Mycoplasma hyopneumoniae modulates ciliary function and epithelial integrity in air-liquid interface porcine respiratory epithelial cells (ALI-PRECs)
Source: Microbiol Spectr. 2026 Jun 18;14(8):e01998-25. doi: 10.1128/spectrum.01998-25 (PMC13435718; doi:10.1128/spectrum.01998-25)
Supplement: Supplementary file 1 [file spectrum.01998-25-s0004.docx]

**Supplemental material**

**Fig. S1.** **Dose- and exposure time-dependent *Mhp* adherence in ALI-PRECs.** ALI-PRECs were mock-inoculated or exposed to *Mhp* 232 strain at doses of 10^5.84^ CCU/ml, 10^6.84^ CCU/ml, or 10^7.84^ CCU/ml. Following 2 h and 5 h of exposure time, the inoculum was removed, transwells were washed, and cells cultures were incubated at 37°C, 5% CO_2_ until fixation with 4% paraformaldehyde at 24, 48, 72 hpi. IFA was performed to detect *Mhp*-P46 membrane protein (FITC). Representative images show P46 detection over time in *Mhp*-inoculated ALI-PRECs. Graph was generated using Fiji Image J v2.14.0/1.54f (NIH) and GraphPad Prism v10.2.1 (GraphPad, San Diego, CA, USA). Scale bar: 50 μm.

**Fig. S2.** **Dose- and time point-dependent loss of integrity in *Mhp*-inoculated ALI-PRECs.** The integrity of *Mhp*-inoculated ALI-PRECs was assessed microscopically using IFA with DAPI cell nuclear staining. Infection medium served as the mock control. (A-B-C) Quantitative analysis of cell nuclei from micrographs captured at 100 x magnification, using Fiji Image J v 2.14.0/1.54f software (NIH). Significant difference in the adjusted number of cells among different doses (ANOVA, $p$ = 0.002), and across various time points (ANOVA, $p$ = 0.019), are shown in (A) and (B), respectively. (C) No significant difference in the adjusted number of cells among exposure time was detected. Graphs and statistical analysis were generated using R Core Team v 4.3.3 (R Foundation for Statistical Computing, Vienna, Austria).

**Fig. S3. Biological variability in loss of integrity in *Mhp*-inoculated ALI-PRECs.** Immunofluorescence (IFA) staining of cell nuclei (DAPI cell nuclear staining) was performed to assess the loss of the integrity of ALI-PRECs overtime. Micrographs captured at 100 x from four symmetric fields were used to perform a multiple comparison among groups. ALI-PRECs from three biological replicates were included. Images were analyzed using Fiji Image J ver. 2.14.0/1.54f software (NIH). Cell nuclei count in *Mhp*-inoculated ALI-PRECs was normalized to mock-inoculated counterparts. (A) Significant difference in the adjusted number of cells among different pigs (ANOVA, $p$ = 0.008) were detected. (B) No significant difference in the adjusted number of cells across time points was detected. Graphs and statistical analysis were generated using R Core Team v 4.3.3 (R Foundation for Statistical Computing, Vienna, Austria) and GraphPad Prism software v10.2.1 (GraphPad, San Diego, CA, USA).

**Movie S1. Morphological changes associated with cytopathic damage in mock-inoculated and *Mhp*-inoculated ALI-PRECs at 72 hpi.**

**Movie** **S2. Temporal assessment of the ciliary movement in mock-inoculated and *Mhp*-inoculated ALI-PRECs over 144 hpi. Scale bar: 50 μm.**

**Movie S3. Temporal assessment of the cytopathic effect in mock-inoculated and *Mhp*-inoculated ALI-PRECs over 144 hpi.**

**Movie S4. Immunofluorescence (IFA) staining for *Mhp*-P46 detection and DAPI cell nuclear staining in *Mhp*-inoculated ALI-PRECs at 24 hpi.**
